# Coding Constraints Modulate Chemically Spontaneous Mutational Replication Gradients in Mitochondrial Genomes

**DOI:** 10.2174/138920212799034802

**Published:** 2012-03

**Authors:** Hervé Seligmann

**Affiliations:** National Collections of Natural History at the Hebrew University of Jerusalem, Jerusalem 91404; Department of Life Sciences, Ben Gurion University, 84105 Beer Sheva, Israel

**Keywords:** Frameshift, overlapping genetic code, protein synthesis, secondary structure formation, RNA synthesis, tRNA, transcription, translation.

## Abstract

Distances from heavy and light strand replication origins determine duration mitochondrial DNA remains singlestranded during replication. Hydrolytic deaminations from A->G and C->T occur more on single- than doublestranded DNA. Corresponding replicational nucleotide gradients exist across mitochondrial genomes, most at 3rd, least 2^nd^ codon positions. DNA singlestrandedness during RNA transcription causes gradients mainly in long-lived species with relatively slow metabolism (high transcription/replication ratios). Third codon nucleotide contents, evolutionary results of mutation cumulation, follow replicational, not transcriptional gradients in *Homo*; observed human mutations follow transcriptional gradients. Synonymous third codon position transitions potentially alter adaptive off frame information. No mutational gradients occur at synonymous positions forming off frame stops (these adaptively stop early accidental frameshifted protein synthesis), nor in regions coding for putative overlapping genes according to an overlapping genetic code reassigning stop codons to amino acids. Deviation of 3rd codon nucleotide contents from deamination gradients increases with coding importance of main frame 3rd codon positions in overlapping genes (greatest if these are 2^nd^ position in overlapping genes). Third codon position deamination gradients calculated separately for each codon family are strongest where synonymous transitions are rarely pathogenic; weakest where transitions are frequently pathogenic. Synonymous mutations affect translational accuracy, such as error compensation of misloaded tRNAs by codon-anticodon mismatches (prevents amino acid misinsertion despite tRNA misacylation), a potential cause of pathogenic mutations at synonymous codon positions. Indeed, codon-family-specific gradients are inversely proportional to error compensation associated with gradient-promoted transitions. Deamination gradients reflect spontaneous chemical reactions in singlestranded DNA, but functional coding constraints modulate gradients.

## INTRODUCTION

The study of replicational gradients, gradual changes in frequencies of specific types of mutational substitutions and the resulting nucleotide contents along genomes [1-2], presents the advantage that evidence originating from bioinformatic comparative genome analyses can be considered as compelling for a mutational gradient and existence of the associated replication origin [3-8]. Gradients result from the fact that different regions of the genome remain singlestranded during different durations, and that DNA is particularly sensitive to certain types of mutations (mainly hydrolytic deaminations A->G and C->T) in the singlestranded state [9-12]. DNA is single stranded during each RNA and DNA synthesis [13-15]. Gradients coincide in each case with distances from promoters of RNA synthesis and replication origins [11, 16-17]. The relative dominance of each, transcriptional or replicational gradient, depends on specific properties of the organism (see discussion and references in [18]). In vertebrate mitochondria, the unidirectional replication of the heavy strand (hs) DNA is initiated at the origin of heavy strand replication (ORIh) [19]. Times spent single stranded by the heavy strand during replication are also determined by the distance of DNA sites from the light strand (ls) replication origin (ORIl), which determines when the hs DNA hybridizes with the freshly synthesized complementary light strand DNA, the double stranded state protecting DNA against mutations [20, 21]. Association of deamination gradients in mitochondria with replication origins is shown by exceptional genomes possessing strand inversions of the replication origin where gradient directions are also inverted [22, 23]. Similarly, phylogenetic comparisons between different species enabled to estimate mutational frequencies in different genes and show that these correspond with singlestrandedness during mitochondrial replication [20, 24]. Results from phylogenetic reconstructions correspond to expectations based on chemical information: the chemically relatively fast C->T reaction associates with a high slope which saturates quickly, as detected by phylogenetic analyses in the mutational gradient, while the slower A->G chemical reaction corresponds to a slower linear gradient across the whole mitochondrial genome [20, 24].

 The study of mutational gradients in mitochondria contributed to the controversy that several heavy strand DNA regions function as light strand replication origins (mainly tDNA templating for tRNAs), not only the recognized ORIl: strengths of gradients starting in the vicinity of tRNA genes are proportional to capacities of the corresponding heavy strand tDNA to form ORIl-like secondary structures [21] and with hs sequences corresponding to the tRNA’s anticodon loop resembling the loop of the regular ORIl in that species [25]. In addition, the stability of hs tDNA: ls tRNA hybridization, presumably competing with ORIl formation by self-hybridization of the hs tDNA, decreases the strength of the corresponding gradient [26]. Comparative analyses of gradients in different primates show that some tRNA genes function as replication origins, while others usually do not [21]. Analyses of human polymorphisms in tRNA genes show that in tRNAs that are not predicted to function as ORIl in the regular physiology, pathogenic mutations tend to increase formation of ORIl-like secondary structures by the hs tDNA, as compared to non-pathogenic polymorphisms: pathogenicity in this case is promoted by increased abnormal ORIl activity, presumably causing deamination gradients such as to promote mutations in genes maladapted to be at the higher end of the deamination gradient. For tRNA genes that comparative analyses suggest to function as ORIl in regular circumstances, the opposite is observed: pathogenic mutations decrease formation of ORIl-like secondary structures, as compared to non-pathogenic polymorphisms [27], meaning that decreased ORIl activity is pathogenic in these cases, perhaps because decreasing rates of replication initiation. Patterns of associations between pathogenicity and hs tDNA: ls tRNA hybridization stabilities also follow these predictions [26]. These analyses indicate that tRNA genes function as ORIl, and contribute to the controversy that multiple light strand replication origins may function in mitochondria [28-30], but the issue is still far from solved and probably more complex than believed, with a number of types of replication processes occurring each at different instances [31, 32].

ORIl formation by hs tDNA in the vicinity of the regular ORIl increases developmental stability as estimated by morphological symmetry in bilateral morphological traits of lizards [33], probably by decreasing durations until hs replication is initiated. ORIl function of hs tDNA distant from the regular ORIl increases the similarity between the replicational and the transcriptional gradient in vertebrate mitochondria. This convergence decreases numbers of genes undergoing high mutational rates according to at least one of these two gradients [18]. Correspondingly, morphological symmetry and lifespan increase with the similarity between replicational and transcriptional gradients, presumably because fewer genes are exposed to high mutation rates. This similarity seems highest in species with long lifespan and slow metabolism, suggesting relatively high transcription/replication ratios.

Gradients result from spontaneous chemical processes, and are strong enough to affect coding properties of genes, as shown by different base and codon usages of genes on leading and lagging strands, which are affected by different mutational pressures due to the deamination gradients [34-36]. The gradients are also themselves affected by coding properties of genomes [37]: for example, while nucleotide contents follow fairly well predictions from replicational gradients in primate mitochondria at 3rd codon positions of protein coding genes (transitions do not result in amino acid replacements at the protein level at the 3rd codon position in vertebrate mitochondria and are hence relatively free to mutate), gradient strengths are weaker at the 1st, and weakest at the 2nd (most crucial codon position from a coding point of view) codon positions [21]. This means that functional constraints related to protein coding and synthesis affect mutational gradients. Several additional factors, beyond codon position, and potentially modulating deamination gradients, are explored here: replication versus transcription, codon-family specific effects on gradients, effects of transitions at 3rd codon positions on codon-anticodon mismatches and protein synthesis accuracy [38, 39], off frame stop codons terminating early frameshifted nonsense protein synthesis after accidental ribosomal slippage [40, 41], and additional coding by overlapping genes [42]. Analyses here are effectuated, when technically possible, on 3rd codon position nucleotide contents of human mitochondrial protein coding genes and on mitochondrial synonymous mutations observed within human populations at 3rd codon positions.

## TRANSCRIPTIONAL VERSUS REPLICATIONAL GRADIENTS: NUCLEOTIDE CONTENTS 

Asymmetry in substitutions associated with time spent single stranded and favouring deaminations results in greater A->G and C->T substitutions on the heavy strand, proportionally to time spent singlestranded. Replicational time spent single stranded (Dsshr) is a function of the distance of a given site from the ORIh and the ORIl. It is Dsshr = 2x(b-ORIl)/N for all genes besides ND1 and ND2, and Dsshr = 2x b/N, where b is the position of the site along the genome numbering from the ORIh, ORIl is the position of the light strand origin of replication according to that positioning convention, and N is the genome length [21, 26]. Transcriptional time spent single stranded is Dssht=b/N [18]. Analyses presented here focus on nucleotide contents of the human mitochondrial genome (NC 012920). Nucleotide contents and mutations in *Homo sapiens* are analysed at the level of the expressed light strand DNA, which means C/T and A/G ratios should increase with duration spent singlestranded. Nucleotide contents result from the cumulation, over evolutionary times, of mutation processes; mutations as these are observed in human populations, are an instantaneous picture of the process. Note that this differs from mutation rates inferred from phylogenetic reconstructions, that include effects of evolutionary time scales on mutations (i.e. [20, 24]) and do not estimate the process at the “moment” it occurs. Fig. (**1**) presents nucleotide ratios at 3rd codon positions, calculated for each protein coding gene from its sequence, as a function of the replicational (filled symbols, continuous lines), respectively transcriptional (hollow symbols, dashed lines) durations spent single stranded for the human mitochondrial genome. Times spent single stranded during replication are better predictors of 3rd codon nucleotide content ratios than those during transcription, for both ratios. Multiple regression analyses show that adding transcriptional durations to replicational ones as predictor of nucleotide ratios does not significantly improve the explained amount of variation in nucleotide ratios (multiple R = 0.76 (A/G) and R = 0.46 (C/T) versus r = 0.70 and r = 0.27 for the replicational gradient alone). Hence the result of evolutionary cumulation of mutation gradients is best explained by replicational processes in *Homo*.

## TRANSCRIPTIONAL VERSUS REPLICATIONAL GRADIENTS: MUTATION RATES

In order to estimate transition rates from variation within human populations, I used the compilations of human mutations from Mitomap (http://www.mitomap.org/MITOMAP, [43]) completed with polymorphisms from mtDB (http://www.mtdb.igp.uu.se, [44]). This procedure estimates more or less instantaneous rates of mutation, rather than the evolutionary cumulation of mutations reflected by nucleotide contents. Fig. (**2**) presents the proportion of 3rd codon positions where heavy strand A->G and G->A substitutions have been observed in human populations. Because annotations usually follow the light strand DNA, the corresponding proportions of each T->C and C->T substitutions on the light strand are shown in Fig. (**2**). Data in Fig. (**2**) show a number of interesting observations. First, gradients exist for T->C, as expected by heavy strand deamination of A->G, but also for C->T. Strengths of these gradients are comparable, which does not fit with predictions from the chemistry of these transitions, the deamination-associated gradient should be stronger. In addition, for T->C, data suggest a stronger transcriptional than replicational gradient, while a weak opposite tendency exists for C->T substitution rates. However, one observation does fit with the known chemistry of pyrimidine transitions: the overall light strand rates of T->C (corresponding to the heavy strand A->G deamination) are higher than those observed for C->T. It is the properties of the gradient itself, other than its intercept, that do not comply with expectations when “instantaneous” mutation rates are examined.

**Fig. (3):**
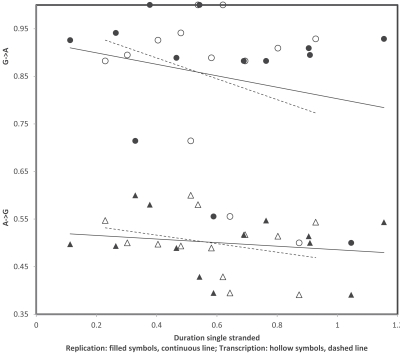
Rates of A->G (triangles) and G->A (circles) transitions at 3rd codon positions on the light strand of human mitochondrial protein coding genes. A gradient according to the heavy strand deamination C->T is expected for light strand G->A. Replicational and transcriptional gradients are indicated by continuous and dashed lines, respectively. Pearson correlation coefficients are: r = -0.19 and r = -0.29 (A->G, replication and transcription, respectively); and r = -0.24 and r = -0.29 (G->A, replication and transcription, respectively).

Similar analyses of transition rates for purines (Fig. **3**) confirm higher overall light strand rates for G->A (corresponds to heavy strand C->T deamination) than for light strand A->G, however each replicational and transcriptional gradients, for both transitions, indicate statistically non-significant trends that are opposite to those expected according to times spent single stranded, independently of whether these would be replicational or transcriptional: substitution rates decrease with singlestrandedness in this case. I suspect that this difference between pyrimidines and purines suggests that an additional process causing mutations is involved, potentially substitutions due to errors by the vertebrate mitochondrial gamma DNA polymerase, but this new potential phenomenon and how it interacts with gradients of singlestrandness will have to be examined in the future.

These results contrast with analyses in Fig. (**1**) of nucleotide contents. This suggests a number of possibilities. It is possible that recent changes in life histories of human populations could have increased the impact of transcription on mutations, which has not yet affected nucleotide contents, but does affect mutation rates in modern times. For example, it is probable that an increased lifespan and older age at reproduction of females result in a greater integration of transcriptional mutations than during most of the previous human evolution. This hypothesis could be tested by similar analyses comparing within-species variation in species with shorter lifespans, and for which lifespan has not recently changed as drastically as in *Homo sapiens*. Another possibility is that nucleotide contents, as well as mutation rates inferred from phylogenetic reconstructions [20, 24], because they integrate the cumulative result of evolution, might be affected by unknown selective pressures that preferentially weed out mutations due to transcription, while those fitting more a replicational gradient are less selected against. In this case, data that fit more evolutionary time scales might reflect more replication, while more instantaneous rates of mutation transcription, because natural selection had less time to affect the latter. This might occur if gene arrangements are determined in relation to their nucleotide contents to minimize impacts of replicational gradients, but not of transcriptional gradients.

In addition, one should note that using evolutionary inferences [20, 24], gradients in transversions are also detected, and these could, by interactions, affect rates of transitions, a further issue to be examined in the future using data from within-species variation, as well as polymerase-associated analyses. A model based on dipole moments of nucleotides suggests that substitution frequencies of nucleotides are proportional to the decrease in the dipole moment due to the substitution, where the general tendency is that nucleotides with high dipole moment tend to be replaced by nucleotides with low dipole moments [45]. This simplistic hypothesis is purely physico-chemical and would reflect only spontaneous forces affecting DNA contents. It is possible that times spent single stranded interact with the dipole moment potential, and this differently for different types of nucleotide substitutions. This potential interaction should also be investigated at some ulterior stage.

## CODON-SPECIFIC DEAMINATION GRADIENTS IN NUCLEOTIDE CONTENTS AT 3RD CODON POSITION

The above section suggests that additional processes affect substitution rates, to the extent that deamination gradients cannot be detected for heavy strand C->T deamination when using mutations as observed in human populations. Though according to the vertebrate mitochondrial genetic code, transitions at 3rd codon positions do not involve amino acid replacements at the protein level, and are hence synonymous mutations, synonymous mutations are not necessarily neutral. These can for example affect mRNA secondary structure formation by self-hybridization, which in many cases preferentially embeds in stems codons that code for conserved (assumed functionally important) amino acids, as opposed to codons in hairpin loops [20, 24, 46, 47]. This phenomenon is believed to protect preferentially against mutations regions that code for functionally important amino acids at protein level. Some processes may be codon-specific, such as the property of error compensation of tRNA misacylation by adequate codon-anticodon mismatch [38-39]. This property varies among synonymous codons and potentially could affect strengths of gradients, according to whether the deamination gradient promotes the synonymous codon with greater or lesser error compensation, which ultimately affects the accuracy of protein synthesis.

In order to investigate the latter possibility, gradient strengths, as estimated by Pearson correlation coefficients, were calculated for each pair of codons from a given codon family and differing at 3rd codon position by a transition. Because this procedure separates in different analyses data for different codon families, data available in terms of mutations observed within human populations are insufficient for analyses, hence only analyses of nucleotide contents were done. These analyses code unity for the light strand nucleotide promoted by the heavy strand deamination gradient (A and C) and zero for the other nucleotide (G and T), and correlation coefficients between these binary variables and times spent single stranded according to each replication and transcription were calculated for the corresponding sites. There are potentially 14 such gradient analyses for purines and 16 for pyrimidines, but for the former, data were insufficient for one pair of codons ending by A or G (CGA/G, which codes for arginine). Table **1** presents the Pearson correlation coefficients estimating the replicational and transcriptional deamination gradients for each codon pair. First, it is notable that the majority of gradients are positive, as expected by the principle of deaminations and single strandedness during replication/transcription. Note that these analyses exclude codons from the gene ND6, which is coded on the strand opposite to other genes: the heavy strand gradient for codons from that gene does not correspond to gradients for the same codons but from other genes.

For heavy strand A->G deaminations (light strand T->C transitions), replicational gradients were positive in 12 among 16 cases (75%, P = 0.0192 according to a one tailed sign test), transcriptional gradients were positive in 10 among 16 cases (62.5%, which is not statistically significant according to a one tailed sign test (P = 0.1136)). Replicational gradients were more positive than transcriptional ones in 11 among 16 cases, P = 0.0525. These results are in line with results indicated by Figs. (**1** and **2**) and confirm that replication is the major process affecting this deamination gradient, also at the level of single codon families.

 For heavy strand C->T deaminations (light strand G->A transitions), transcriptional gradients were positive in 11 among 13 cases (85%, P = 0.007 according to a one tailed sign test), but only in 46% of the cases for replicational gradients (which does not differ from 50% expected by chance). Transcriptional gradients were more positive than replicational ones in 62% of the cases. The tendency is positive and statistically significant according to a sign test for transcriptional gradients, but there is no apparent trend for replicational gradients, confirming that these data fit better a transcriptional than replicational gradient, as indicated by Figs. (**1** and **3**) for this deamination gradient.

## DEAMINATION GRADIENTS AND PATHOGENIC MUTATIONS

**Fig. (4):**
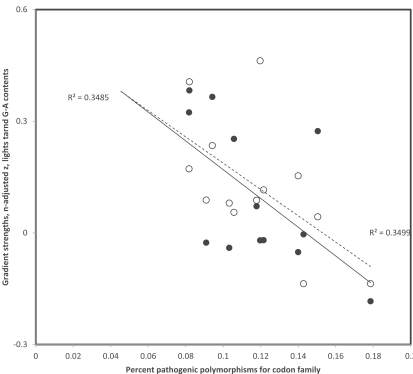
Third codon position gradient strengths (sample size adjusted z of Pearson correlation coefficient r) of codon pairs with A and G at 3rd codon position as a function of percentage of pathogenic polymorphisms in that codon family. Data are from Table **1**. Filled and hollow circles are for replicational and transcriptional gradients, respectively (one tailed P = 0.0169 and P = 0.0165, respectively). Percentages of pathogenic polymorphisms, as estimates of selection against mutations and associated gradients due to deamination mutations, affect similarly transcriptional and replicational gradients.

**Fig. (5):**
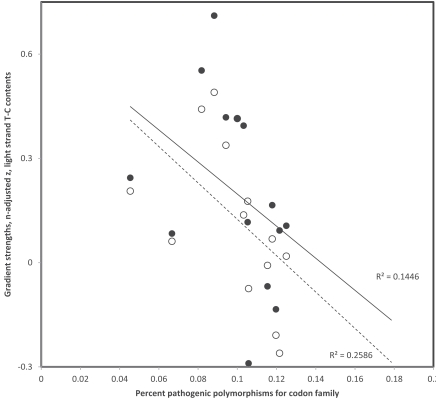
Third codon position gradient strengths (sample size adjusted z of Pearson correlation coefficient r) of codon pairs with T and C at 3rd codon position as a function of percentage of pathogenic polymorphisms in that codon family. Data are from Table **1**. Filled and hollow circles are for replicational and transcriptional gradients, respectively (one tailed P = 0.0899 and P = 0.0317, respectively). Percentages of pathogenic polymorphisms, as estimates of selection against mutations and associated gradients due to deamination mutations, affect more transcriptional than replicational gradients.

Data in Table **1** suggest that variation exists among codon families and between purines and pyrimidines in strengths of deamination gradients. It is possible that selection weakens more the gradient for some codons than for others. If this is correct, an estimate of selection specific to each codon family may be expected to be inversely proportional to gradient strength. For each codon family investigated, the percent of polymorphisms that are associated with pathologies was calculated from the two last columns of Table **1**, assuming that this percentage indicates selection on synonymous mutations in that codon family. Because gradient strengths are calculated on different numbers of data (column 3 in Table **1**), and that sample sizes affect estimates of Pearson correlation coefficients r, these r values which estimate gradient strengths were z transformed using the adjustment method of r for sample size effects used in [48]: z(adjusted)=z - r/(2n-5), where z is the regular z transformation of r, z = log((1+r)/(1-r)) and n is the sample size. For codon pairs with G-A at 3rd position, corresponding to heavy strand deamination gradients of C->T, strengths of z-adjusted replicational and transcriptional gradients (data from Table **1**) decrease with percentages of pathogenic mutations found for that codon family (r = -0.590 and r =-0.592, respectively, see Fig. **4**). For codon pairs with T-C at 3rd position, corresponding to heavy strand deamination gradients of A->G, strengths of z-adjusted replicational and transcriptional gradients (data from Table **1**) also decrease with percentages of pathogenic mutations for that codon family (r = -0.380 and r =-0.509, respectively, see Fig. **5**). These results indicate that the propensity of 3rd codon nucleotide contents to follow the tendency due to the spontaneous chemistry of deamination gradients is affected by functional effects of the deamination on codon function. 

In codons where polymorphisms are rarely pathogenic, the gradients are strong, hence synonymous codon positions seem free to follow simple spontaneous chemical tendencies. But the more frequently polymorphisms associate with pathologies, and hence are counter-selected, the weaker the deamination gradients associated with that codon pair: functional constraints prevent the free mutation of 3rd codon positions in accordance with the deamination gradient associated with duration of singlestrandedness because these synonymous mutations often affect functions and hence cause pathologies.

## DEAMINATION GRADIENTS AND ERROR COMPENSATION OF tRNA MISACYLATION BY CODON-ANTICODON MISMATCH

Results in the previous section indicate that functional properties associated with synonymous transitions at 3rd codon position affect gradient strengths. According to a naïve approach, this could be considered as surprising, because such transitions are not supposed to affect proteins. However, 3rd codon positions, though not altering coding contents, potentially affect the accuracy of coding. Indeed, codon-anticodon mismatches differ between codons that end by either A or G, or U or C. This is important in relation to a property known as error compensation of tRNA misacylation by codon-anticodon mismatch [38-39]. 

This process of error compensation results in correct amino acid insertion into the protein sequence, according to the amino acid coded by the codon. It consists of the adequate combination of codon-anticodon mismatches and tRNA misacylations. It is estimated by the Pearson correlation coefficient r between misacylation tendencies of tRNAs (using the online application tfam, http://tfam.lcb. uu.se, [49]) and the stability of the duplex interaction (DINAMelt, http://mfold.rna.albany.edu/?q=DINAMelt/Two-state-melting, [50, 51]) between the tRNA’s anticodon and the codon coding for the misacylated amino acid. The use of tfam to estimate misacylation and error compensation have each been previously discussed [52, 38]. Error compensation can be calculated for specific tRNA anticodons as well as codons. Because numbers of codon-anticodon mismatches vary among codons and anticodons, the adjusted z transformation of Pearson’s correlation coefficient r is also used here, as done in a previous section for estimates of gradient strengths. Here, codon-specific error compensation is calculated for human mitochondrial genomes, as done previously [38].

**Fig. (6):**
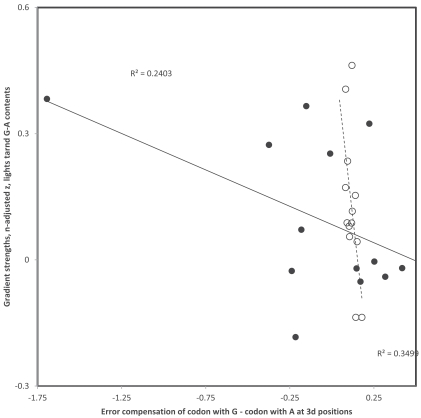
Third codon position gradient strengths (sample size adjusted z of Pearson correlation coefficient r) of codon pairs with A and G at 3rd codon position as a function of difference in error compensation between codons with G and A at 3rd codon position in that codon family (error compensation is also a sample size adjusted z of a Pearson correlation coefficient). Data are from Table **1**. Filled and hollow circles are for replicational and transcriptional gradients, respectively (one tailed P = 0.0445 and P = 0.0165, respectively). Error compensation is greater in the codon promoted by the deamination gradient the more negative the x axis is. The negative trend indicates that strong gradients occur when the error compensation is greater in the codon promoted by the gradient.

Error compensation may be relevant to 3rd codon position deamination gradients specific to codon pairs differing by a transition at the 3rd codon position in the following way: the gradient will be strong if the error compensation of the codon promoted by the gradient is greater than that of the other synonymous codon. Hence, according to the heavy strand C->T deamination gradient, the gradient will be strong in codon pairs where error compensation is greater in the light strand codons ending with A than those ending with G, and weak when the opposite is the case. The above stated predictions are verified, mainly for the association between transcriptional gradients and differences of error compensation between codons from the same codon family, but ending with A, versus those ending with G (Fig. **6**). Similarly, according to the heavy strand A->G deamination gradient, the gradient should be strong in codon pairs where error compensation is greater in light strand codons ending with C than those ending with T, and weak when the opposite is the case. Fig. (**7**) shows data consistent with this prediction, though more weakly than for the previous gradient type, but in this case too mainly for transcriptional gradients. Hence error compensation is confirmed as one of the functional constraints affecting the spontaneous chemistry of deaminations. If deaminations decrease error compensation, therefore promoting amino acid misinsertions, substitutions due to that gradient are counterselected and no (or a weak) gradient is observed. When the deamination promoted by the 3rd codon position gradient increases error compensation, hence promoting accuracy of protein synthesis, the gradient observed for that codon pair is strong.

## OFF FRAME STOPS AND DEAMINATION GRADIENTS

In addition to regular in frame coding properties such as those described in previous sections, 3rd codon transitions can affect also functional properties related to off frame functions in the gene. The presumed function consists in off frame stop codons stopping early protein synthesis after accidental ribosomal slippage. The heavy strand deamination gradient of A->G at 3rd codon positions (of the regular open reading frame) has the potential to destroy UAA and UAG stops existing in the +2 frame of genes, transforming them into CAA and CAG, which would not stop frameshifted protein synthesis. Fig. (**8**) shows that light strand C contents at 3rd codon positions (of the regular open reading frame) of human mitochondrial genes increases with replicational times spent single stranded for codons that cannot participate in the formation of +2 frameshifted UAA or UAG stop codons. However, for those codons (of the regular open reading frame), in the same genes, that do form +2 frameshifted stops UAA or UAG, or potentially could participate after a transition (+2 frameshifted CAA or CAG codons), the gradient does not exist or is very weak: light strand C contents, which is promoted by the heavy strand A->G deamination gradient, does not increase in the context that it would destroy +2 frameshifted light strand stops UAA or UAG. The trends in Fig. (**8**) show that 3rd codon position C contents (of the regular open reading frame) in contexts potentially forming these off frame stops is systematically lower than for the rest of the codons ending with C or T in that gene.

The same principle can be applied for the heavy strand C->T deamination, corresponding to G->A transitions on the light strand. This gradient potentially promotes formation, according to context, of +1 frameshifted UAA or UAG stops from UGA or UGG off frame codons, and of the +2 frameshifted AGA or AGG stops from GGA or GGG off frame codons. For +1 frameshifted AGA and AGG stops, this deamination gradient would promote the destruction of off frame stops into AAA or AAG off frame codons.

For +1 frameshifted UAA or UAG stop codons, and UGA or UGG codons that could form a stop after a transition at 3rd codon position (of the regular open reading frame), the deamination promoted by the heavy strand C->T gradient would increase off frame stop codon densities. Fig. (**9**) plots 3rd codon A contents (of the regular open reading frame) in genes as a function of replicational times spent singlestranded, separating codons that do not or cannot, according to context, contribute to formation of +1 frameshifted UAA or UAG stops (filled circles, continuous line), and those codons who do or could contribute to such stops after transition at 3rd codon position of the regular open reading frame (hollow circles, dashed line). A gradient exists for codons (of the regular open reading frame) that do not have this specific off frame stop function. No gradient (or a much weaker one) is found for those off frame codons who do have that function or potentially could have it. In the latter case, A contents at 3rd codon positions (of the regular open reading frame) are greater than expected by the spontaneous chemistry of the deamination gradient in the rest of the codons in the gene. This suggests that deaminations that form stops are promoted not by the gradient in times spent single stranded, but by the functional advantage of possessing an off frame stop, independently of the position of the gene along the gradient, and far beyond levels expected by the deamination gradient.

**Fig. (8):**
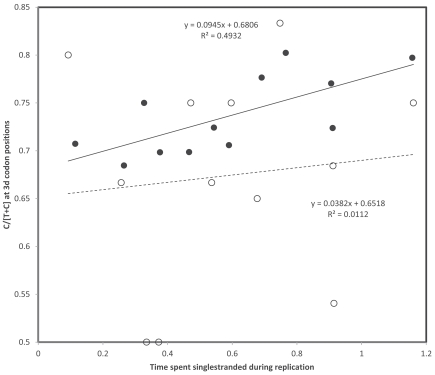
Third codon nucleotide contents for codons with T or C at 3rd codon position in human mitochondrial genes as a function of replicational time spent single stranded by that gene, separating from other codons those forming +2 frameshifted UAA or UAG and CAA or CAG codons. The heavy strand deamination gradient A->G increases the light strand contents of C in codons that do not participate in formation of UAA or UAG +2 frameshifted stops (filled circles, continuous line), but this is not the case when the codon context enables formation of such off frame stops (hollow circles). In the latter case, deaminations seem counterselected and C is rare at 3rd codon positions (as shown by the low and statistically non-significant slope of the dashed line). This case fits functional predictions of the off frame stop hypothesis. ANCOVA shows the constants of the two lines differ significantly (one tailed P = 0.042).

**Fig. (9):**
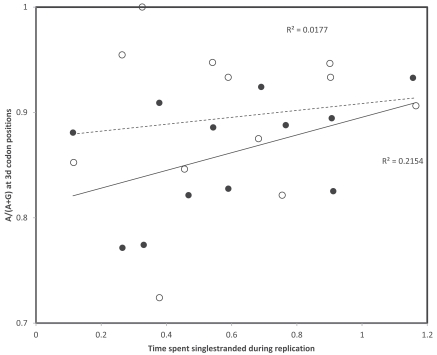
Third codon nucleotide contents for codons with A or G at 3rd codon position in human mitochondrial genes as a function of replicational time spent single stranded by that gene, separating from other codons those forming +1 frameshifted UAA or UAG and UGA or UGG codons. The heavy strand deamination gradient C->T increases the light strand contents of A in codons that do not participate in formation of UAA or UAG +2 frameshifted stops (filled circles, continuous line), but this is not the case when the codon context enables formation of such off frame stops (hollow circles). In the latter case, deaminations seem favoured beyond levels promoted by the spontaneous chemistry of the replication gradient and A is usually more common at 3rd codon positions (dashed line) than predicted by the replicational gradient (continuous line), as shown by the low and statistically non-significant slope of the dashed line. This case fits functional predictions of the off frame stop hypothesis. ANCOVA shows the constants of the two lines differ significantly (one tailed P = 0.0035).

A similar situation exists for +2 frameshifted AGA or AGG stop codons, and GGA or GGG codons that could form a stop after a transition at 3rd codon position. The deamination promoted by the heavy strand C->T gradient at 3rd codon position of the regular open reading frame would increase off frame stop codon densities. Fig. (**10**) plots 3rd codon A contents (of the regular open reading frame) in genes as a function of replicational times spent singlestranded, separating codons that do not or cannot, according to context, contribute to formation of +2 frameshifted AGA or AGG stops (filled circles, continuous line), and those codons who do have that function or could contribute to such stops after transition at 3rd codon position of the regular open reading frame (hollow circles, dashed line). A gradient exists for codons (of the regular open reading frame) that do not have this specific off frame stop function, but does not or is much weaker for those who do or potentially could have this off frame stop function. In the latter case, A contents at 3rd codon positions (of the regular open reading frame) are greater than expected by the spontaneous chemistry of the deamination gradient in the rest of the codons in the gene, suggesting that deaminations that form stops are promoted beyond levels of the gradient in times spent single stranded. Potentially, this would be by the functional advantage of possessing an off frame stop, independently of the position of the gene along the gradient, as for the two previous cases (Figs. **8**, **9**).

The last analysis of this type relates to +1 frameshifted AGA or AGG stop codons, and AAA or AAG codons that could form stops after a transition at 3rd codon position of the regular open reading frame. The deamination promoted by the heavy strand C->T gradient at 3rd codon position of the regular open reading frame would decrease off frame stop codon densities. Fig. (**11**) plots 3rd codon position A contents (of the regular open reading frame) in genes as a function of replicational times spent singlestranded, separating codons that do not or cannot, according to context, contribute to formation of +1 frameshifted AGA or AGG stops (filled circles, continuous line), and those codons who do or could contribute to such stops after transition at 3rd codon position of the regular open reading frame (hollow circles, dashed line). In the latter case, A contents at 3rd codon positions (of the regular open reading frame) are greater than expected by the spontaneous chemistry of the deamination gradient in the rest of the codons in the gene, suggesting that deaminations that destroy stops are promoted beyond levels of the gradient in times spent single stranded. This result does not fit with the prediction that the functional advantage of possessing off frame stops affects deamination rates and the associated gradients, it is actually opposite to prediction. This case differs from the three previous ones presented in this section.

**Fig. (11):**
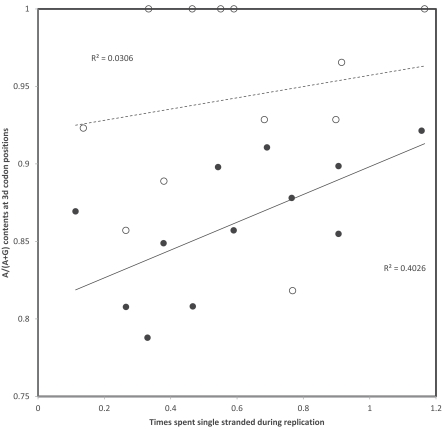
Third codon nucleotide contents for codons with A or G at 3rd codon position in human mitochondrial genes as a function of replicational time spent single stranded by that gene, separating from other codons those forming +1 frameshifted AGA or AGG and AAA or AAG codons. The heavy strand deamination gradient C->T increases the light strand contents of A in codons that do not participate in formation of AGA or AGG +1 frameshifted stops (filled circles, continuous line), but this is not the case when the codon context enables formation of such off frame stops (hollow circles). In the latter case, deaminations seem favoured beyond levels promoted by the spontaneous chemistry of the replication gradient and A is usually more common at 3rd codon positions (as indicated by the weak and statistically nonsignificant slope of the dashed line) than predicted by the replicational gradient (continuous line). This case does not fit functional predictions of the off frame stop hypothesis. ANCOVA shows the slopes of the two lines differ significantly (two tailed P = 0.01).

**Fig. (12):**
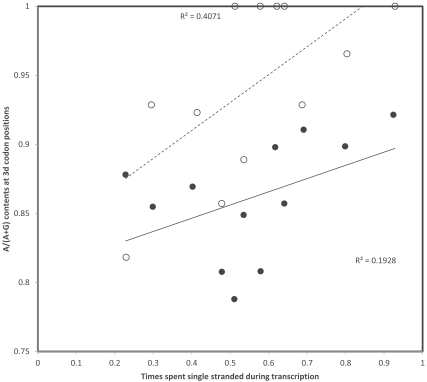
Third codon nucleotide contents for codons with A or G at 3rd codon position in human mitochondrial genes as a function of transcriptional time spent single stranded by that gene, separating codons forming +1 frameshifted AGA or AGG and AAA or AAG codons. The heavy strand deamination gradient C->T increases the light strand contents of A in codons that do not participate in formation of AGA or AGG +1 frameshifted stops (filled circles, continuous line), but this is not the case when the codon context enables formation of such off frame stops (hollow circles). In the latter case, deaminations seem favoured beyond levels promoted by the spontaneous chemistry of the replication gradient and A is usually more common at 3rd codon positions (dashed line) than predicted by the replicational gradient (continuous line). This case does not fit functional predictions of the off frame stop hypothesis. It is the only case where 3rd codon position contents of codons participating in off frame stops follow a deamination gradient. ANCOVA shows the slopes of the two lines differ significantly (two tailed P = 0.0002).

The case of effects of +1 frameshifted AGA or AGG stops on the heavy strand deamination gradient promoted by the transition C->T (Fig. **11**) differs also from the other three cases presented in Figs. (**8-10**) in another respect. In all three analyses corresponding to Figs. (**8-10**), effects fitted functional expectations of the ambush/off frame stop codon hypothesis and were clearest for replicational gradients, and weakest for transcriptional gradients (not shown). For the analyses of +1 frameshifted AGA or AGG stops presented in Fig. (**11**), a qualitative difference in favour of transcriptional gradients exists (Fig. **12**). This analysis considering transcriptional times spent single stranded does not resolve the conundrum that data do not fit expectations of the off frame stop functional hypothesis. However, a strong transcriptional deamination gradient fitting the spontaneous chemistry of deamination exists for 3rd codon position contents of codons participating in formation of these +1 frameshifted AGA or AGG stops, stronger than for the other codons. In all previous analyses, 3rd codon position contents of codons participating in formation of off frame stops do not fit deamination gradients, and are either (almost) systematically above or below the regular gradient, and this according to the adaptive rationale of maximizing off frame stop codon numbers. In the cases of Figs. (**8-10**), their relative positions in relation to the regular gradient suggests that off frame stops are promoted beyond or against the gradient, according to the simple functionally adaptive rationale of promoting densities of off frame stops. For each replicational and transcriptional analyses in Figs. (**11**, **12**), stops are more prevented than predicted by the regular gradient at positions not involved in off frame stop formation. But for the transcriptional gradient in Fig. (**12**), the gradient is clearly stronger for codons participating (or potentially participating) in off frame stop codon formation than for those who do not. Hence preventing off frame stop formation occurs at levels beyond the gradient, forming an enhanced gradient. This suggests unknown and unsuspected functional constraints promoting deaminations in this specific codon context, and in a way interacting with the deamination gradient. 

It is possible that this puzzling observation related to avoidance of +1 frameshifted AGR mitochondrial stop codons relates to the peculiar way these codons function as termination codons (in the regular coding main frame), by causing -1 frameshifts that, when the AGR codon is preceded by U, creates a UAG stop codon [53], but further recent observations on vertebrate mitochondrial termination suggest that we are still far from understanding this atypical mode of termination of translation [54].

## OVERLAP CODING GENES AND DEAMINATION GRADIENTS

Besides off frame stop codons, other off frame functions exist that could interact with deamination gradients at 3rd codon positions. Approximately 30% of the total length of regular main frame mitochondrial protein coding genes participates in same-strand overlap coding of additional protein coding genes [42]. Similarly, about 30% of the total protein coding gene length participates in overlap coding on the complementary, antisense strand. These overlapping genes include “stop” codons in their putative coding sequences, a fact that at first would suggest that these putative proteins are not expressed in mitochondria. However, mitochondria frequently import cytosolic tRNAs, which include tRNAs with anticodons recognizing AGA and AGG codons and with cognate Arg. These codons associate with termination of translation in vertebrate mitochondria [53], but import of these cytosolic tRNAs would switch on the expression of these overlapping genes. Similarly, bioinformatic analyses predict in some mitochondrial genomes the existence of antisense tRNAs that possess anticodons matching both UAR and AGR stop codon families (antisense antitermination tRNAs, [55]). The predicted number of overlapping genes and their contents in “stops” associates positively with the predicted presence of antisense antitermination tRNAs (recognizing AGR codons) as well as capacities to form cloverleaf secondary structures for tRNAs recognizing UAR stops [42].

These overlapping genes have potential impact on deamination gradients at 3rd codon positions, in a way similar, in principle, to that exposed in the previous section for off frame stops. In regions of regular main frame protein coding genes participating in overlap coding, 3rd codon positions in the regular main frame would be less free to mutate according to the deamination gradient because in the overlapping gene, the nucleotide usually does not correspond to a 3rd codon position. Hence 3rd codon position nucleotide contents in these regions should not (or much less) correspond to deamination gradients than for regions of the same genes, but where no overlap coding occurs.

Figs. (**13** and **14**) plot 3rd codon position nucleotide contents corresponding to each the heavy strand C->T and A->G deaminations as a function of time spent single stranded during replication, separating between codons coding only in the main, regular open reading frame (filled symbols), and those coding also in at least one other frame (open symbols). Results for both gradients show that 3rd codon positions coding only in the regular main frame follow the replicational deamination gradient, however, this is not or much less the case for regions contributing to coding in other frames. These appear as much more constrained, which prevents nucleotide contents to follow the purely chemical process of deamination, because of coding properties of these nucleotides in other frames. This result concurs with the existence of additional coding frames within the regular open reading frames. Deamination gradients have already been used for the detection of otherwise undetected genes [56], and the example here shows their use for confirming the possible existence of overlapping genes in mitochondria.

## OVERLAP CODING INTENSITY AND DEAMINATION GRADIENTS

The analyses in Figs. (**13**, **14**) plot separately nucleotide contents of overlapping gene regions when different overlapping frames, for a given regular main gene, are involved in overlap coding, and when more than one overlapping gene exists for that sequence. For example, there are in total 5 datapoints for the gene ND1, two representing its 5’and 3’ extremities which each code only in the main frame. The three additional ones correspond to different types of overlap coding regions. A first region corresponds to overlap coding in the +2 frame of the same strand as the regular open reading frame, which means that the 3rd codon position of the regular main frame is in this case also the first codon position in the +2 frameshifted overlapping gene. The third datapoint in this group corresponds to overlap coding in the third frame of the complementary (or antisense) strand of the gene. In that case, the 3rd codon position of the main frame corresponds to the second codon position in the overlapping gene on the complementary strand.

There is a region between the two latter regions that codes in the +2 frame of the same strand as the main regular reading frame, and in the 3rd frame of the complementary strand. Over the whole genome, there were in total 5 regions coding in 3 frames, 12 in 2 frames, and 26 coding only in the regular main frame.

The nucleotide content data analysed here correspond always to the 3rd codon position in the regular main frame. This is because this codon position is most free to mutate because transitions are synonymous at this position. When first codon positions are analysed, the gradient is weaker, because transitions at that position affect more proteins, as these alter the coded amino acid, however, such mutations usually replace amino acids by other amino acids that are relatively similar in physico-chemical properties, leading to relatively conservative changes at the protein level. Therefore, some of these mutations can be tolerated, and some effects of deamination gradients can be observed also at first codon positions, though these are less clear than at 3rd codon positions. Transitions at 2^nd^ codon positions have a strong effect at protein level, because they will result in amino acid replacements that differ widely in physico-chemical properties, and hence will be almost always counterselected. As a result, nucleotide contents at 2^nd^ codon positions do almost not reflect deamination gradients.

For the 3rd codon positions of the regular main frame that participate in overlap coding, their coding properties in the overlapping gene should affect nucleotide contents in relation to the deamination gradient, and this according to whether the 3rd codon position of the regular main frame is predicted to function as a 1st codon position in the overlapping gene (this occurs when the overlapping gene is in the +2 frame of the same strand, and for the first coding frame of the complementary strand), a 2nd codon position in the overlapping gene (this occurs when the same strand overlapping gene is in the +1 frame, and for the 3rd coding frame of the complementary strand), or a 3rd codon position in both genes (this occurs only for the 2nd coding frame on the complementary strand). This means that the effect of overlap coding on nucleotide contents, in terms of deviation from the regular deamination gradient observed for regions with regular non-overlap coding, depends on the type of overlap coding, with the least effects when the position is the 3rd codon position in both coding frames. Extents of deviation from the gradient are estimated by the residuals from the regression fitting the gradient. The coding importance of the position (that functions as 3^rd^ codon position in the regular main frame) was semi-quantitatively estimated for the overlapping gene the following way: 1 when it is predicted to function also as 3rd codon position in the overlapping gene, 2 when predicted to function as 1st codon position in the overlapping gene, and 3 when predicted to function as 2nd codon position in the overlapping gene. Hence a 3rd codon position participating in overlap coding in the +2 same strand frame has coding importance 1+2=3. Its coding importance is 1+3=4 if it is a +1 same strand frame overlap gene. A 3rd codon position participating in same strand +2 frame and in complementary strand frame 3 coding, has coding importance 1+2+2=5. Such coding importance estimations were done for each coding region, according to the coding frame(s) detected by previous analyses [42]. 

**Fig. (13):**
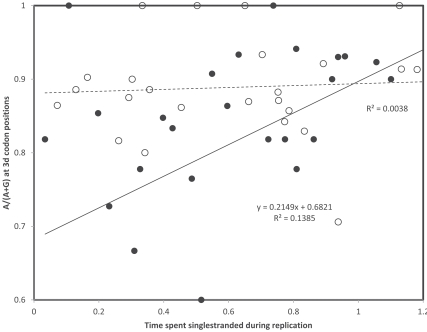
Nucleotide content at 3rd codon positions for purines (A or G) as a function of duration spent singlestranded during replication, separating codons coding only in the regular main frame of the protein coding gene (filled symbols), and those coding also in another frame, for an overlapping gene (on the same or on the complementary strand, hollow symbols), as described elsewhere [42]). The phenomenon corresponds to the heavy strand replicational deamination gradient due to C->T deaminations and is clearly visible for 3rd codon positions that code only in the regular open reading frame. Codons coding also in other frames, hence participating to other genes, and for which 3rd codon position nucleotide contents are also constrained by coding properties in the overlapping gene, do not respond to the replication gradient.

**Fig. (14):**
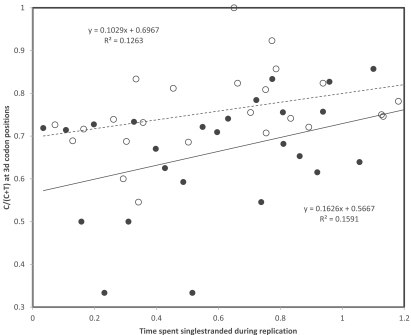
Nucleotide content at 3rd codon positions for pyrimidines (C or T) as a function of duration spent singlestranded during replication, separating codons coding only in the regular main frame of the protein coding gene (filled symbols), and those coding also in another frame, in an overlapping gene (on the same or on the complementary strand, hollow symbols), as described elsewhere [42]). The phenomenon corresponds to the heavy strand replicational deamination gradient due to A->G deaminations. The gradient is clearly steeper for 3rd codon positions that code only in the regular open reading frame. Codons coding also in other frames, hence participating to other genes , and for which 3rd codon position nucleotide contents are also constrained by coding properties in the overlapping gene, respond less to the chemically spontaneous replication gradient.

I used the regression lines calculated between 3rd codon position nucleotide contents (Figs. **13** and **14**) with times spent singlestranded during replication to calculate residual nucleotide contents for each region. These residuals were plotted as a function of the coding importance (calculated as in the examples above) in all frames. These analyses show that deviation, as estimated by the residuals, in nucleotide contents from the deamination gradient increases the greater the coding importance of the (regular main frame) 3rd codon positions in the overlapping gene(s). Coding importance explains 6 and 9% of the unexplained variation in Figs. (**13** and **14**), respectively. However, the different nucleotide contents are calculated on the basis of varying numbers of codons, because the different regions vary widely in lengths. Hence a large part of the variation in nucleotide contents can be expected to be due to small sample size effects, rather than to biological, coding properties. A reanalysis of the data, weighing datapoints according to numbers of codons used to calculate nucleotide contents for each datapoint, shows that taking into account sample sizes increases the amount of variation explained by coding importance, to 18 and 29%, respectively, for each residuals from Figs. (**13** and **14**), respectively. Fig. (**15**) plots the residuals averaged for both nucleotide ratios as a function of coding importance of 3rd codon positions for that region. The positive trend shows clearly the effects of coding importance on deviations from deamination gradients, especially after weighted regression analysis integrates effects of sample sizes. These results do not only show effects of overlap coding on deamination gradients. They show that an explicit model that includes information on the coding function of the nucleotides in the overlapping genes (their position in the overlap codon) explains how much the gradient can or cannot affect nucleotide contents in these coding regions. The fact that explicit coding information from the overlapping genes explains nucleotide contents in deamination gradients is a strong independent confirmation of existence of overlap coding in vertebrate mitochondrial genomes.

## GENERAL DISCUSSION

The analyses presented here integrate several factors into the exploration of deamination gradients. All show that replicational deamination gradients are affected by functional constraints. In several cases, purine and pyrimidine deamination gradients differ qualitatively in this respect, which justifies analysing separately these two phenomena. Note in this context that anticodon nucleotide contents of tRNAs reflect transcriptional deamination gradients [27] for each purines and pyrimidines, while 3rd codon position analyses shown here tend to reflect replication constraints for pyrimidines, and transcription gradients for purines. 

The discrepancies between analyses based on nucleotide contents and mutation rates inferred from phylogenetic comparisons, as opposed to those from direct, nearly instantaneous observations of mutations in human populations, in terms of fitting replication versus transcriptional gradients, stress the fact that long term genomic phenomena are not simple cumulative results of short term processes, because various unknown additional factors shape the long term outcome. Hence phylogenetic as well as single species analyses should be repeated on different taxonomic groups, and compared, considering the evolutionary depths of the comparisons, as well as the life histories of the taxa. Such analyses are likely to reveal further useful information on molecular mechanisms.

It is important to remember that not all mutations are due to deaminations or other spontaneous chemical reactions. Some are due to errors done by the mitochondrial DNA gamma polymerase. It would be valuable to investigate these effects on deamination gradients, especially that times spent single stranded might interact with polymerase fidelity, because these durations also reflect the age of the replication fork.

A further issue that is worth investigating in the future is the choice of the analysis scale, not only in terms of evolutionary time scale as noted above, but also in terms of spatial scale at the level of the genome. At first, an approach using natural units as done here seems preferable, which means either site by site analyses, or averaging sites according to genes, which are natural functional units. However, it is possible that varying sampling window sizes between these two extremes might reveal aspects of the phenomenon of deamination gradients that are not detectable otherwise. Previous analyses also showed that secondary structure formation (by DNA or RNA) might decrease deamination rates at the level of single sites [20, 24, 46], a phenomenon worth noting and exploring further.

As also noted previously [18], it might be important to invest efforts into learning to differentiate between gradients due to transcription versus replication (especially if sometimes replication mimicks transcription), in terms of gradient slopes, and relative effects on C->T versus A->G deaminations. This issue remains also open for the future.

Different mitochondrial genome regions apparently gain and lose the function of light strand replication origin [57]). This seems especially frequent for heavy strand DNA coding for tRNAs. Hence the joint analysis of deamination gradients and formation capacities of ORIl-like structures by heavy strand tDNA could show the dynamics of evolutionary gain and loss of a second function, an endeavour of interest for adaptive analysis of evolution of acquisition of gene functions.

The results presented in the various sections here include a number of surprises, but the major one is that relatively simple analyses of nucleotide contents of a single genome yield a plethora of adaptively coherent results. Analyses of gradients at the codon family level are such an example, and show how even supposedly subtle functional constraints specific to the codon family, related to the accuracy of codon-anticodon interactions, can affect whole genome nucleotide contents. Previous studies of tRNA sequences in relation to ORIl-like function showed that a single substitution affecting formation of ORIl-like structures by the tDNA can alter patterns of replication initiation, and through the resulting deamination gradients, the distribution of nucleotides across the whole genome, and possibly also the need for adequate gene rearrangement to decrease negative impacts of the deamination gradients on the coding properties of the genes [58]. The analyses of error compensation for synonymous codons shows that a single substitution at the tRNA level, because it potentially alters the error compensation profile of that tRNA by affecting its recognition by tRNA synthetases and hence misacylation probabilities, can as a result affect deamination gradients at the level of the whole genome. If there is one message to remember from this study of deamination gradients, it is that genomes are highly integrated: many processes can make that in many cases, an apparently small change at a very local scale, and with supposedly unrelated properties, can be the effector of significant large scale changes at genome level.

The importance of off frame coding properties is also underlined by analyses presented above. These show that many processes and properties of genes and genomes remain unknown despite efforts spent studying them. In this respect, all examples show that taking into account the deamination gradients, and exploring the additional processes potentially shaping the genome (synonymous codon usage, off frame stops, overlap coding) in an integrated way proved very rewarding, in the sense that in most cases, most observations were congruent with expectations developed from simple working hypotheses, while the fewer exceptions open the way to new ideas and investigations, reminding us that we are unaware of many aspects of the processes at work. 

One of the important aspects contributing to this relative success is that the deamination gradient functions in the analyses presented here as a null hypothesis predicting a specific pattern, against which to compare patterns from data in relation to other adaptive processes associated with different functional hypotheses. But it is not only the statistical power of the experimental design that enabled the detection of effects of the various processes. Theoretically, one could have thought that error compensation of tRNA misacylation is a quantitatively marginal phenomenon, especially when comparing synonymous codons. However the results presented here show that this is not the case, and this supposedly rare event actually shapes the distribution of synonymous codon usages. The same could have been thought of the issue of off frame stop codons. Presumably, ribosomal slippages are rare, and the increased efficiency due to early stopping of framshifted protein synthesis at off frame stop codons could be assumed as weak. Nevertheless, analyses presented here detect strong effects in relation to this, suggesting the phenomenon is more important than suspected, which is in accordance with observations that off frame stop codon densities increase developmental stability in lizards [59]. This might be also because frameshifted protein synthesis requires strict regulation, considering that numerous off frame overlapping genes apparently occur within the regular protein coding genes [42], as also confirmed here by detailed analyses of deamination gradients in relation to overlapping genes. These results stress that in addition to unknown processes, the relative importance of many known processes and phenomena has frequently been underestimated. These can be relevant to phenotypes, as also suggested by associations with pathogenic mutations (i.e. Figs. **4** and **5**).

Mitochondrial sequences are frequently used for phylogenetic reconstructions. Such analyses are frequently based on evolutionary models for DNA sequence changes, which assume neutrality, and do not account for phenomena such as deamination gradients, which affect the mutational probabilities, and hence likelihoods of phylogenetic reconstructions. Results presented here also stress that in addition to the importance of accounting for gradients in mutation probabilities, a large number of sites, even at 3rd codon positions, cannot evolve freely, because numerous functional constraints, such as off frame stops and overlap coding affect them. Hence, while efforts aimed at reconstructing phylogenetic histories of taxa, and the use of these phylogenies to explore for the evolution of natural history traits is highly laudable, I reiterate here my cautionary warning [60] that phylogenetic models seem still too incomplete to integrate on biologically realistic terms the various processes underlying evolutionary changes of DNA.

The study of the relatively simple mitochondrial genomes is not only important for phylogenetic reconstructions. It has already shown its importance as a model for replication and repair of nuclear genomes [61]. The recent discovery that off frame sequences code for previously undetected protein coding genes according to a new, different genetic code that reassigns stop codons to amino acids [42] shows that the study of mitochondrial genomes could probably also revolutionize our understanding of coding and the versatility of the genetic code, also because such overlap coding by an overlapping genetic code probably also occurs in nuclear genomes. In the context of this major discovery, the analyses of deamination gradients presented here, which include explicit information on overlap coding, are an important independent confirmation that large parts of the mitochondrial genome are involved in overlap coding. This underlines the importance of the analysis of deamination gradients as a useful, simple tool to help reveal additional, new molecular phenomena that escaped our attention until now.

## Figures and Tables

**Fig. (1):**
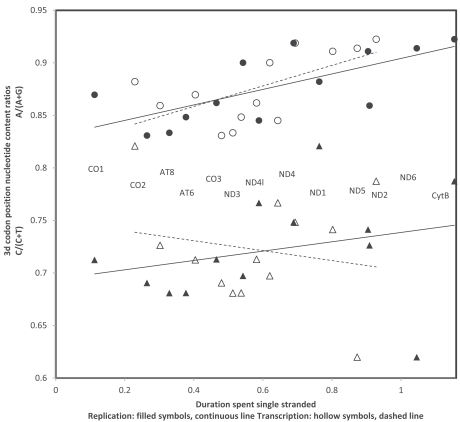
Nucleotide content ratios at 3rd codon positions as a function of times spent single stranded for the 13 mitochondrial protein coding genes in *Homo sapiens*. Gene identities are indicated according to their positions according to the replicational gradient. Circles are for the A/(A+G) ratio, triangles for the C/(C+T) ratio. Pearson correlation coefficients are: r = 0.70 and r = 0.61 (A/(A+G), replication and transcription, respectively); and r = 0.27 and r = -0.19 (C/(C+T), replication and transcription, respectively).

**Fig. (2):**
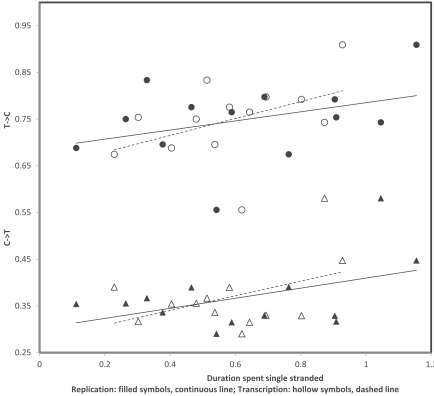
Rates of C->T (triangles) and T->C (circles) transitions at 3rd codon positions on the light strand of human mitochondrial protein coding genes. A gradient according to the heavy strand deamination A->G is expected for light strand T->C. Replicational and transcriptional gradients are indicated by continuous and dashed lines, respectively. Pearson correlation coefficients are: r = 0.36 and r = 0.44 (T->C, replication and transcription, respectively); and r = 0.46 and r = 0.43 (C->T, replication and transcription, respectively).

**Fig. (7):**
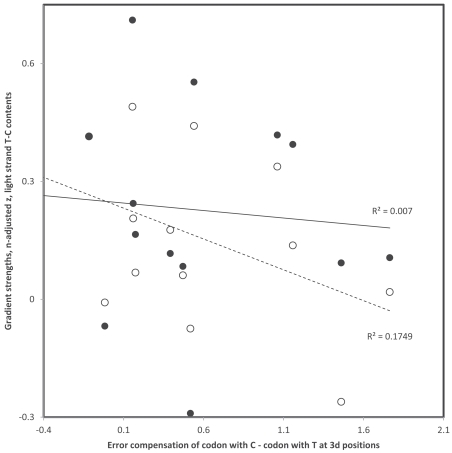
Third codon position gradient strengths (sample size adjusted z of Pearson correlation coefficient r) of codon pairs with T and C at 3rd codon position as a function of difference in error compensation between codons with C and T at 3rd codon position in that codon family (error compensation is also a sample size adjusted z of a Pearson correlation coefficient). Data are from Table **1**. Filled and hollow circles are for replicational and transcriptional gradients, respectively (one tailed P = 0.3876 and P = 0.0684, respectively). Error compensation is greater in the codon promoted by the deamination gradient the more negative the x axis. The negative trend indicates that strong gradients occur when the error compensation is greater in the codon promoted by the gradient.

**Fig. (10):**
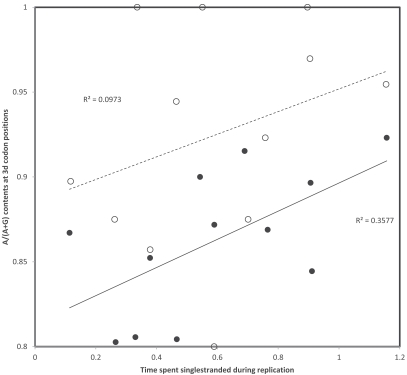
Third codon nucleotide contents for codons with A or G at 3rd codon position in human mitochondrial genes as a function of replicational time spent single stranded by that gene, separating from other codons those forming +2 frameshifted AGA or AGG and GGA or GGG codons. The heavy strand deamination gradient C->T increases the light strand contents of A in codons that do not participate in formation of AGA or AGG +2 frameshifted stops (filled circles, continuous line), but this is not the case when the codon context enables formation of such off frame stops (hollow circles). In the latter case, deaminations seem favoured beyond levels promoted by the spontaneous chemistry of the replication gradient and A is usually more common at 3rd codon positions (as indicated by the weak and statistically nonsignificant slope of the dashed line) than predicted by the replicational gradient (continuous line). This case fits functional predictions of the off frame stop hypothesis. ANCOVA does not detect statistically significant differences between the two lines.

**Fig. (15):**
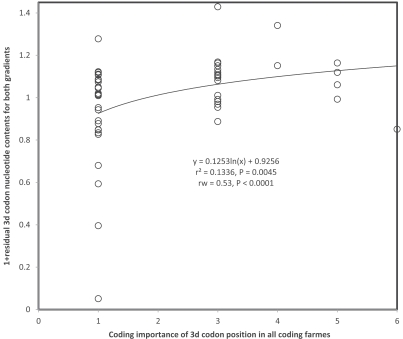
Residual 3rd codon position nucleotide content averaged for data from Figs. (**13** and **14**) (+1 to avoid negative values), as a function of the coding importance of the 3rd codon position in relation to overlapping genes. 3rd codon position coding only in non-overlapping regions gets coding importance “1”, the coding importance in overlapping genes is added to this (coding importance for 1st codon position is 2, 3 for 2nd position).

**Table 1.** Codon-Specific Deamination Gradients and Error Compensation of tRNA Misacylation by Codon-Anticodon Mismatch

Amino acid	Codon	N	Repl	Trans	Error1	N1	Error2	N2	Pol	Pathos
Lys	AAA-G	83-10	-0.026	0.077	0.418	4	0.468	5	50	7
Thr	ACA-G	130-10	-0.010	0.058	-0.384	8	-0.182	10	181	22
Met	AUA-G	165-32	0.136	0.022	-0.068	13	-0.252	17	113	17
Gln	CAA-G	82-8	-0.013	0.44	0.265	9	0.146	10	44	4
Pro	CCA-G	52-7	-0.010	0.228	-0.123	6	-0.043	8	142	17
Leu	CUA-G	277-42	0.036	0.044	0.354	10	0.266	15	399	47
Glu	GAA-G	63-15	0.190	0.201	0.505	8	-0.315	9	61	5
Ala	GCA-G	82-5	0.181	0.117	0.327	10	0.253	12	138	13
Gly	GGA-G	62-18	-0.02	0.040	0.121	9	0.279	9	155	16
Val	GUA-G	61-8	0.126	0.028	0.297	15	0.292	18	104	11
Ser	UCA-G	81-7	0.161	0.086	0.676	8	0.729	10	110	9
Trp	UGA-G	89-9	-0.002	-0.068	-0.289	19	-0.168	20	70	10
Leu	UUA-G	65-9	-0.092	-0.069	-0.038	12	-0.146	17	84	15
Asn	AAC-U	131-29	0.042	0.031	-0.722	9	-0.593	7	90	6
Thr	ACC-U	153-49	0.046	-0.130	-0.552	7	0.154	5	181	22
Ser	AGC-U	38-10	-0.034	-0.004	0.456	10	0.445	13	26	3
Ile	AUC-U	194-114	0.053	0.009	-0.433	11	0.420	9	200	25
His	CAC-U	79-18	0.122	0.103	-0.027	8	0.057	10	44	2
Pro	CCC-U	120-36	-0.067	-0.104	-0.99	2		1	142	17
Arg	CGC-U	26-6	0.342	0.241	0.277	15	0.350	12	34	3
Leu	CUC-U	168-64	0.085	0.034	-0.584	6	-0.532	5	399	47
Asp	GAC-U	51-12	0.062	-0.015	-0.579	13	-0.250	11	39	2
Ala	GCC-U	124-40	0.206	0.168	0.152	9	0.608	8	138	13
Gly	GGC-U	87-16	0.195	0.069	0.158	17	0.635	14	155	16
Val	GUC-U	46-22	-0.145	-0.038	0.122	15	0.369	13	104	11
Tyr	UAC-U	89-35	-0.111	-0.076	0.592	13	-0.243	11	89	9
Ser	UCC-U	99-28	0.270	0.218	-0.066	5	0.247	4	110	9
Cys	UGC-U	17-4	0.207	0.207	0.013	18	-0.046	17	10	1
Phe	UUC-U	139-69	0.058	0.088	-0.539	6	-0.376	6	114	12

Column 1 indicates the amino acid coded by the codon pair indicated in column 2, N are numbers of codons for that codon pair in all human mitochondrial protein coding genes, excluding ND6 because it is coded by the heavy strand DNA, columns 4-5 indicate replicational, respectively transcriptional gradient strengths for that codon pair (estimated by Pearson correlation coefficients r), columns 6-9 indicate error compensation for that codon pair (estimated by r) and numbers of codon-anticodon mismatches involved in that calculation, column 10 is the number of codons presenting polymorphisms for that codon family, and column 11 is the number among these that associate with pathologies.
